# Mucinous Adenocarcinoma of the Appendix: The Challenges of Managing a Complex Surgical Case

**DOI:** 10.7759/cureus.20157

**Published:** 2021-12-04

**Authors:** Nirav Vyas, Mamun Dornseifer, Manoj Nair

**Affiliations:** 1 General Surgery, North Middlesex University Hospital, London, GBR

**Keywords:** hyperthermic intraperitoneal chemotherapy, appendicitis, cytoreductive surgery, hiv, mucinous adenocarcinomas

## Abstract

Mucinous adenocarcinomas of the appendix are rare and often present as a suspected appendicitis. Diagnostic work-up encompasses colonoscopy, tissue biopsy, CT scan, and a multidisciplinary team input. Management involves surgery, hyperthermic intraperitoneal chemotherapy, and adjuvant chemotherapy. Our patient was known to be human immunodeficiency virus (HIV) positive; therefore, careful consideration had to be taken in starting adjuvant chemotherapy as there were concerns of drug interactions and further immunosuppression. Despite all these challenges the patient has had an excellent outcome with no evidence of recurrence or distant disease.

## Introduction

Mucinous adenocarcinomas (MAA) of the appendix are extremely rare, constituting less than 0.5% of all gastrointestinal tract neoplasms [1]. These neoplasms are challenging to manage as they can rupture and produce peudomyxoma peritonei. The mean age of presentation is 60 years and there is no increase in risk with either sex [2]. Patients normally present with symptoms similar to appendicitis. Therefore, good diagnostic work-up is needed to ensure a correct diagnosis is made. This will involve a CT scan looking for collections and possible metastasis. Once the patient is stable, a tissue diagnosis is essential with a colonoscopy. MAA can cause extensive disease within the peritoneum; therefore, treatment modalities would include cytoreductive surgery with hyperthermic intraperitoneal chemotherapy (HIPEC) and adjuvant therapy. Our brief review of a case presentation of a 58-year-old male with a diagnosis of MAA aims to highlight the clinical presentation, diagnostic work-up, and treatment approaches in achieving a desirable outcome.

## Case presentation

A 58-year-old man presented to the general surgical team with a one-week history of constant right iliac fossa pain radiating to his back. He did not report any other concerning symptoms - weight loss or change in bowel habit. Previous clinic letters indicated he had intermittent colicky right iliac fossa pain 6 years prior, which was investigated with a CT abdomen and no abnormality was detected. His past medical history included hypertension and human immunodeficiency virus (HIV) stage 3, which is the most severe stage of HIV infection where individuals are most immunocompromised [3]. He was taking antiviral medication for his HIV with a good CD4 count. His past surgical history included bilateral inguinal hernia repair. He denied any family history of malignancies.

The bed-side abdominal examination demonstrated a soft abdomen with tenderness and guarding in the right iliac fossa, and no masses were palpable. Biochemistry revealed an elevated white cell count of 10.02 × 10^9^/L and a C-reactive protein of 133.7 mg/dL, and the remainder of his blood results were unremarkable.

**Figure 1:**
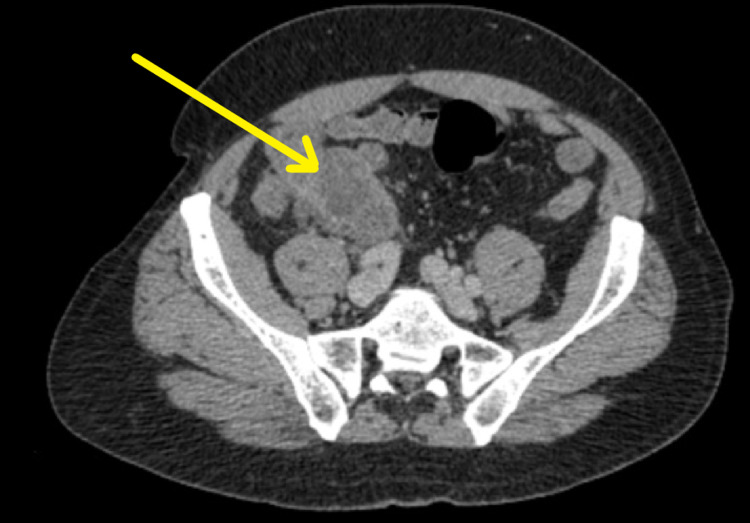
CTAP on admission CTAP: CT abdomen and pelvis

CT abdomen and pelvis with contrast revealed an appendiceal abscess (Figure 1). This finding was discussed at the local X-ray multidisciplinary team (MDT) meeting, where a decision was made to manage it conservatively with broad-spectrum antibiotics and CT-guided drainage. This improved his symptoms substantially and was discharged two days later with a drain in-situ which was to be reviewed as an outpatient. Cytology from the drainage did not reveal any malignant cells.

Following his discharge, he was further investigated with an outpatient colonoscopy, which revealed a caecal lesion and biopsy showed a high-grade dysplasia. A repeat CT scan still demonstrated a para appendiceal collection, which had improved. 

In view of these findings he was referred to Basingstoke Hospital, a tertiary centre. He was reviewed at Basingstoke and a decision was made for him to undergo cytoreductive surgery. Subsequently the patient underwent complete cytoreduction surgery with HIPEC. He lost approximately 400 mL of blood during the surgery and did not require any blood products. Following his surgery he was transferred to the intensive care unit.

He made a good recovery post-operatively and was eventually discharged without any post-operative complications. Post-operatively his tumour was staged T4a N0 M0 (0/13 ileocolic nodes, 0/1 omental nodes) and a CT scan showed no recurrence. Following his recovery he underwent adjuvant therapy with capecitabine, with close input from the HIV team.

Two years since his initial presentation and having completed all his treatments, our patient is alive and in remission. He does not currently suffer from any complications from his treatments. We continue to see him in our surveillance programme.

## Discussion

Given the patient's age, above the age of 45, an initial investigation of a CT scan is appropriate. A CT abdomen and pelvis with contrast, on admissions, revealed a well-defined marginally enhancing collection in the right iliac fossa measuring 6.4 by 4.5 cm related to the medial aspect of the caecal pole with multiple adjacent lymph nodes (Figure 1). These image findings were consistent with an acute appendiceal abscess. 

A top differential diagnosis in a male presenting with right iliac fossa pain and raised inflammatory markers is appendicitis. Appendiceal malignancies are commonly misdiagnosed as appendicitis [4]. Other differentials that should be considered are terminal ileum Crohn's disease, hernias, and, as in this case, malignancies. In female patients gynaecological causes should also be considered.

In our case the initial CT scan ruled out other bowel pathology. At this point we had to differentiate between a simple abscess formed by infective causes or a malignancy - as this would dictate the management. The only way to be able to differentiate would be from a tissue biopsy.

A diagnosis of high-grade MAA was achieved based on histology from the colonoscopy specimens. A staging CT was able to identify there was no evidence of metastatic disease. With this information a discussion could begin on the appropriate management.

Initial treatment at the time of presentation aims to stabilize the patient. Sepsis six was started immediately while further investigations were performed. Once an appendiceal collection was identified, CT-guided drainage aimed to reduce the size of the collection and therefore reduce the level on infection present. Once the patient was stable this allowed us more time to organize MDT meetings for input of the best investigations moving forward. 

The management pathway for mucinous adenocarcinoma of the appendix can be separated into two based on whether there is a high-grade or low-grade dysplasia [5]. Low grade can be managed with just a resection of the residual disease. However, with high grade, as in this case, complete debulking surgery is recommended with HIPEC [5]. He underwent cytoreductive surgery involving a right hemicolectomy, parietal peritonectomy, omentectomy, and the falicform ligament excision. During the surgery he received 1 hour of HIPEC with mitomycin at 42 degrees Celsius, followed by thorough lavage. There has not been many studies looking into the mortality and complication rates of HIPEC on HIV patients. However, performing HIPEC and cytoreductive surgery on a HIV patient is risky, because previous studies have shown that performing laparotomies on patients with AIDS had an overall mortality of 30% and a complication rate of 46% [6]. 

During the administration of his adjuvant therapy careful consideration had to be taken, with two main concerns being further immunosuppression and drug interactions with his HIV medication. This posed a real challenge administering the adjuvant therapy. A decision was made to give six months of capecitabine with the aim of adding 5% to 10% added benefit in addition to the surgery. He was also started on co-trimoxazole as prophylaxis for pneumocystis pneumonia. Careful monitoring of his CD4 count and toxicity was needed during the treatment. He completed three months of the adjuvant therapy; however, he then developed significant amount of palmar plantar erythema. At this point they felt the risks would outweigh the benefits of continuation of treatment. Therefore, capecitabine was stopped and he had to resume the surveillance programme.

There are a very limited number of case reports on patients with HIV undergoing HIPEC and cytoreductive surgery. However, the most important point these cases emphasise is that patients with a positive HIV status alone should not be denied the clearly indicated surgery [7]. Despite numerous challenges a positive outcome can be achieved. 

## Conclusions

The timeline of this case from presentation to the date of his surgery was five months. This was a difficult case given the MDT approach needed involving numerous teams, the patient being highly immunocompromised and attempting to coordinate his care in the middle of the COVID-19 pandemic, which caused a delay in a lot of patient’s care. Despite these difficulties we can conclude that he was managed efficiently, delivering an ideal outcome as he currently remains in remission.

The main take-away points from this could be the importance of immediate MDT input involving all relevant teams, that adjuvant therapy can be given in patients with a high risk of immunosuppression, and that appendiceal malignancies can easily be misdiagnosed, therefore proper work should always be done when managing appendicitis. It is important to remember that a positive HIV status alone should not be a criterion to deny patients of clearly indicated treatments.
